# An Enhanced ZigBee-Based Indoor Localization Method Using Multi-Stage RSSI Filtering and LQI-Aware MLE

**DOI:** 10.3390/s25092947

**Published:** 2025-05-07

**Authors:** Jianming Li, Shuyan Yu, Zhe Wei, Zhanpeng Zhou

**Affiliations:** 1 School of Computer Science, Civil Aviation Flight University of China, Guanghan 618307, China; lugeeljm@gmail.com (J.L.); findz@cafuc.edu.cn (Z.W.); zhouzhanpeng@cafuc.edu.cn (Z.Z.); 2 Yuanpei College, Shaoxing University, Shaoxing 312000, China

**Keywords:** indoor positioning, ZigBee, RSSI filtering, maximum likelihood estimation, link quality indicator

## Abstract

Accurate indoor localization in wireless sensor networks remains a non-trivial challenge, particularly in complex environments characterized by signal variability and multipath propagation. This study presents a ZigBee-based localization approach that integrates multi-stage preprocessing of received signal strength indicator (RSSI) data with a reliability-aware extension of the maximum likelihood estimation (MLE) algorithm. To improve measurement stability, a hybrid filtering framework combining Kalman filtering, Dixon’s Q test, Gaussian smoothing, and mean averaging is applied to reduce the influence of noise and outliers. Building on the filtered data, the proposed method introduces a noise and link quality indicator (LQI)-based dynamic weighting mechanism that adjusts the contribution of each distance estimate during localization. The approach was evaluated under simulated and semi-physical non-line-of-sight (NLOS) indoor conditions designed to reflect practical deployment scenarios. While based on a limited set of representative test points, the method yielded improved positioning consistency and achieved an average accuracy gain of 11.7% over conventional MLE in the tested environments. These results suggest that the proposed method may offer a feasible solution for resource-constrained localization applications requiring robustness to signal degradation.

## 1. Introduction

In 2000, Hightower et al. [1] proposed a SpotON system. This system utilizes multiple readers, using the target’s received signal strength indicator (RSSI) [2] as a parameter to estimate distance, which is then used to determine the target’s location through triangulation. Although this method has relatively low accuracy, it provides an important reference for subsequent positioning research. Currently, positioning algorithms based on distance measurement primarily include angle of arrival (AOA) [3,4], time of arrival (TOA) [5,6], and time difference of arrival (TDOA) [7]. Among them, positioning methods based on TOA and TDOA rely heavily on the synchronization precision of transmitters and receivers, imposing high requirements on the equipment. Distance measurement methods can also use hyperbolic positioning in addition to geometric triangulation. By deploying three nodes at different locations, two hyperbolas can be constructed based on the difference in distances between the target and two pairs of nodes, and their intersection determines the target’s position. The distance difference can be obtained through TDOA. However, distance-based positioning methods usually require multiple readers, making the system more complex and costly. In complex indoor environments, wireless signal propagation is severely affected, significantly degrading positioning performance.

Given the limitations of traditional distance-based methods such as reliance on multiple readers, hardware complexity, and vulnerability to signal degradation in indoor environments, this study adopts ZigBee as the underlying communication platform. ZigBee offers several advantages for wireless sensor network-based localization, including low power consumption, mesh networking, and native support for both RSSI and link quality indicator (LQI) metrics. Unlike radio frequency identification (RFID), which typically requires external readers and supports only unidirectional communication, ZigBee enables real-time, bidirectional data transmission and flexible network topology. These characteristics allow for continuous data collection and adaptive signal processing, making ZigBee particularly suitable for the implementation of the proposed multi-stage filtering and dynamic weighting strategies. Prior research has also demonstrated ZigBee’s robustness in dense, multipath-prone environments [8,9,10,11,12], supporting its selection as a practical foundation for scalable indoor 3D localization.

To improve positioning accuracy in complex indoor environments, this study proposes an enhanced localization approach that integrates a multi-stage signal preprocessing framework with a reliability-aware estimation model. Specifically, a hybrid Kalman-Dixon-Gaussian-Mean (KDGM) filtering pipeline is introduced to suppress noise and remove outliers in the collected RSSI signals. On this basis, a noise and link quality indicator (LQI) weighted maximum likelihood estimation (NLW-MLE) method is applied, which dynamically adjusts the influence of each ranging value according to its estimated reliability. Measurements with lower noise levels and higher LQI values are assigned greater weights, while those affected by significant interference or link degradation contribute less to the final position calculation.

This approach addresses several limitations observed in conventional localization methods. Traditional centroid and weighted centroid algorithms are known to exhibit large errors in weak signal conditions or when anchor node distribution is uneven. Likewise, standard maximum likelihood estimation MLE [13,14] models typically assume uniform measurement quality, making them sensitive to fluctuations in signal reliability. By incorporating dynamic signal weighting and lightweight preprocessing, the proposed method enhances positioning robustness and estimation accuracy without incurring substantial computational overhead.

Experimental evaluations conducted under simulated and semi-physical non-line-of-sight (NLOS) indoor conditions show that the NLW-MLE algorithm achieves an average improvement of 11.7% in localization accuracy compared to the conventional MLE baseline. These results demonstrate the practical effectiveness of the method for reliable, real-time positioning in complex indoor wireless sensor network environments.

The remainder of this paper is organized as follows. Section 2 reviews the relevant background and existing methods in three-dimensional indoor localization, focusing on the triangular centroid, weighted centroid, and maximum likelihood estimation (MLE) algorithms. Following this, Section 3 introduces the preprocessing techniques adopted to improve RSSI signal reliability, including a multi-stage filtering pipeline that integrates Kalman filtering [15], Dixon’s Q test [16], and Gaussian filtering [17]. In Section 4, the proposed NLW-MLE algorithm is presented in detail, highlighting the incorporation of noise and LQI into a dynamic weighting mechanism to enhance localization accuracy. Subsequently, Section 5 describes the simulation and experiments conducted to evaluate the performance of the proposed method, accompanied by analyses and visualizations of the results. Finally, Section 6 and Section 7 discuss and conclude this study, respectively.

## 2. Background and Related Work

### 2.1. Triangular Centroid Localization Method in 3D Environments

In practical three-dimensional positioning systems, the location of a blind node is typically estimated using distance measurements from multiple anchor nodes with known coordinates. While the trilateration model is a common approach, its performance is often affected by ranging errors and anchor placement inaccuracies. As a result, the ideal scenario where three spheres intersect at a single point rarely occurs. Instead, the intersection forms an overlapping region with spatial uncertainty. To mitigate this effect, the triangular centroid localization method [18] has been introduced, which estimates the position of the blind node by computing the geometric centroid of the three intersection points derived from the overlapping region. This method enhances positioning stability under practical constraints and is especially useful in environments where precise ranging is difficult to achieve.

In three-dimensional space, given the distances $r_{1},\;r_{2}$, and $r_{3}$ between the blind node and three anchor nodes, we can construct three sphere equations to describe the geometric relationships. Let the coordinates of the blind node be $P=\left(x,y,z\right)$, and the coordinates of the anchor nodes be $O_{i}=\left(x_{i},y_{i},z_{i}\right)$, where i=1,2,3 represents the three anchor nodes. The equation for the ith sphere can then be expressed as:

(1) $$∥P-O_{i}{∥}^{2}=r_{i}^{2},i=1,2,3,$$

where $∥P-O_{i}∥$ represents the Euclidean distance between the blind node and the ith anchor node. The expanded form is given by:

(2) $$\left\{\begin{array}{c} \sqrt{(x-x_{1}{)}^{2}+(y-y_{1}{)}^{2}+(z-z_{1}{)}^{2}}=r_{1}\\ \sqrt{(x-x_{2}{)}^{2}+(y-y_{2}{)}^{2}+(z-z_{2}{)}^{2}}=r_{2}\\ \sqrt{(x-x_{3}{)}^{2}+(y-y_{3}{)}^{2}+(z-z_{3}{)}^{2}}=r_{3} \end{array}\right.,$$

These equations represent the three spheres in three-dimensional space, where each sphere is centered at the position of an anchor node, and its radius corresponds to the distance to the blind node. Theoretically, these three equations define the spatial distance relationship between the blind node and the three anchor nodes. If there were no measurement errors, solving this system of equations would directly yield the blind node’s coordinates $\left(x,y,z\right)$.

However, due to practical factors such as ranging errors and device accuracy limitations, the intersection of these three spheres does not converge precisely at a single point but rather forms an overlapping region. Consequently, solving for the intersection points alone is insufficient for accurately determining the blind node’s position.

To mitigate the uncertainty caused by errors in real-world applications, the geometric centroid of the three intersection points is typically used to estimate the blind node’s position. These intersection points are obtained by solving the system of sphere equations. Assuming that the three intersection points of the overlapping region are $A\left(x_{A},y_{A},z_{A}\right)$, $B\left(x_{B},y_{B},z_{B}\right)$, and $C\left(x_{C},y_{C},z_{C}\right)$, the estimated coordinates of the blind node, denoted as $P\left(x_{p},y_{p},z_{p}\right)$, are given by the centroid formula:

(3) $$\left\{\begin{array}{c} X_{p}=\frac{x_{A}+x_{B}+x_{C}}{3}\\ Y_{p}=\frac{y_{A}+y_{B}+y_{C}}{3}\\ Z_{p}=\frac{z_{A}+z_{B}+z_{C}}{3} \end{array}\right.,$$

**Figure 1:**
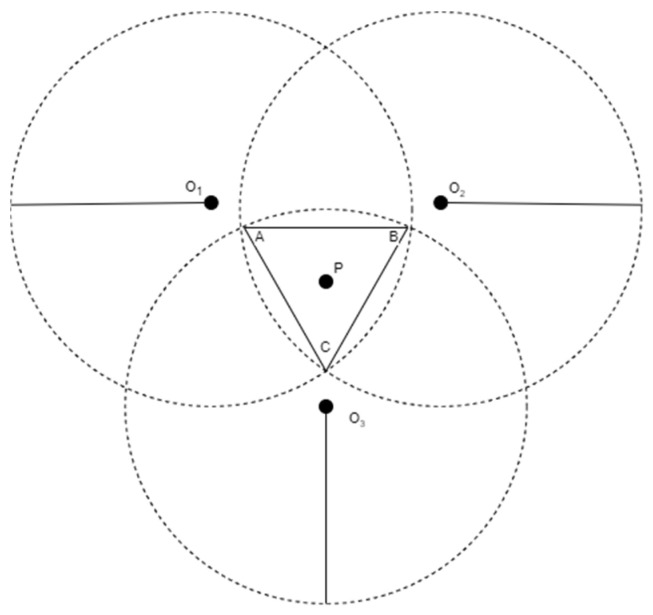
Triangular centroid localization diagram.

As shown in Figure 1, this is a three-dimensional schematic from a two-dimensional perspective, illustrating how the distances between the three anchor nodes and the blind node define three spheres. Ideally, these three spheres would intersect at a single point. However, due to ranging errors, their intersection may form only a finite overlapping region instead of a precise single point.

**Figure 2:**
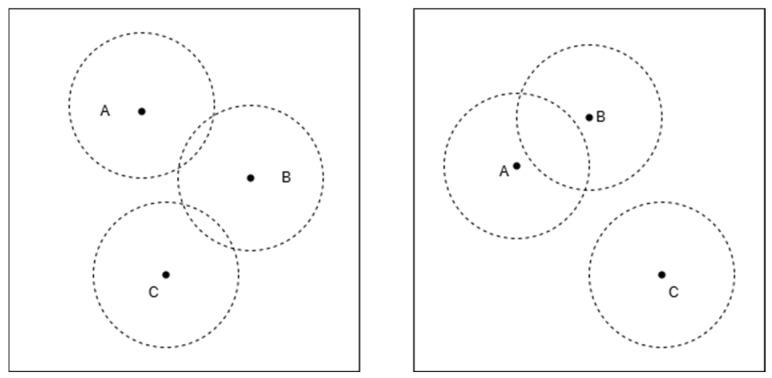
Three-circle intersection without a common overlapping region. A, B, and C are the centers of the three circles, respectively.

**Figure 3:**
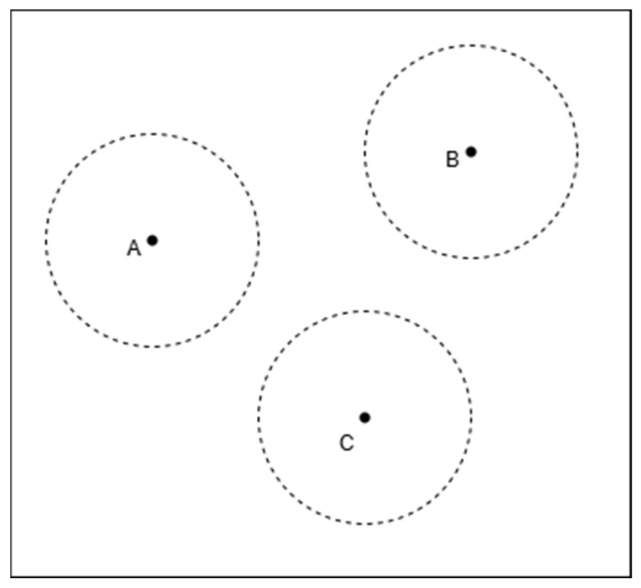
Three-circle non-intersection. A, B, and C are the centers of the three circles, respectively.

Figure 2 and Figure 3 illustrate two typical cases of the intersection of three circles, which may impact the effectiveness of the triangular centroid localization method in practical positioning applications. To gain a deeper understanding of how these scenarios affect positioning accuracy, we will conduct a detailed analysis of each case presented in the figures.

As shown in Figure 2, the three circles have intersections, but their overlapping regions do not merge into a single common area. Although each pair of circles has intersection points, the overall intersection does not form a unique shared region or a single intersection point.

This scenario typically occurs when there are large ranging errors, causing discrepancies in the radii and positions of the circles, leading to a discontinuous or scattered intersection region.

In the three-dimensional triangular centroid localization method, the accuracy of positioning relies on the uniqueness of the intersection region of the spheres. If the three spheres fail to form a common overlapping region, the blind node’s position cannot be accurately estimated using the centroid of the intersection.

As a result, in such cases, centroid-based positioning methods face high uncertainty, which may lead to increased estimation errors in determining the blind node’s location.

As shown in Figure 3, the three circles are completely non-intersecting, with noticeable distances between them. This indicates that the intersection region of the three circles is empty, meaning that the given radii and center positions do not form a common overlapping area. Typically, this situation occurs when ranging errors are excessively large or when the spatial distribution of anchor nodes is inappropriate.

### 2.2. Weighted Triangular Centroid Localization Method

The triangular weighted centroid localization (TWCL) method [19] is an improved version of the traditional triangular centroid localization method, retaining the advantages of low computational complexity, ease of implementation, and minimal communication overhead. Unlike the traditional centroid localization method, TWCL fully considers the differences in signal strength from various beacon nodes to the unknown node, particularly incorporating the impact of RSSI values.

In the traditional centroid localization algorithm, the three beacon nodes are treated equally in their influence on the unknown node, which can introduce positioning errors. The TWCL method improves upon this by assigning different weights to each beacon node based on their respective distances from the target.

Assume that the coordinates of the reference points A, B, and C are given as $A\left(x_{1},y_{1},z_{1}\right)$, $B\left(x_{2},y_{2},z_{2}\right)$, and $C\left(x_{3},y_{3},z_{3}\right)$, respectively. The distances from the target point to these reference points are denoted as $d_{1}$, $d_{2}$, and $d_{3}$. Based on these distances, the weighting formula for each reference point is given by:

(4) $$\omega_{1}=\frac{1}{d_{1}+d_{2}},\omega_{2}=\frac{1}{d_{1}+d_{3}},\omega_{3}=\frac{1}{d_{2}+d_{3}},$$

The rationale behind this calculation method stems from the inverse relationship between distance and signal strength attenuation. In many positioning systems, signal strength is typically inversely proportional to distance. That is, the closer the distance, the stronger the signal and the smaller the error. Therefore, reference points that are closer should be assigned greater weights, while those farther away should have relatively lower weights.

Additionally, considering that each reference point is at a different distance from the target, their contributions to the target’s position should be weighted according to the inverse of the distance. This approach enhances the accuracy of localization by giving more influence to closer reference points.

Using these weights, the target point’s coordinates in three-dimensional space can be computed through a weighted average method. Specifically, the coordinates x, y, and z of the target point are given by the following formulas:

(5) $$\left\{\begin{array}{c} x_{p}=\frac{\left(\frac{x_{1}}{d_{1}+d_{2}}+\frac{x_{2}}{d_{1}+d_{3}}+\frac{x_{3}}{d_{2}+d_{3}}\right)}{\frac{1}{d_{1}+d_{2}}+\frac{1}{d_{1}+d_{3}}+\frac{1}{d_{2}+d_{3}}}\\ y_{p}=\frac{\left(\frac{y_{1}}{d_{1}+d_{2}}+\frac{y_{2}}{d_{1}+d_{3}}+\frac{y_{3}}{d_{2}+d_{3}}\right)}{\frac{1}{d_{1}+d_{2}}+\frac{1}{d_{1}+d_{3}}+\frac{1}{d_{2}+d_{3}}}\\ z_{p}=\frac{\left(\frac{z_{1}}{d_{1}+d_{2}}+\frac{z_{2}}{d_{1}+d_{3}}+\frac{z_{3}}{d_{2}+d_{3}}\right)}{\frac{1}{d_{1}+d_{2}}+\frac{1}{d_{1}+d_{3}}+\frac{1}{d_{2}+d_{3}}} \end{array}\right.,$$

This method calculates the three-dimensional coordinates of the target point using inverse distance weighting, ensuring that reference points closer to the target have a greater influence on the localization result, thereby improving positioning accuracy. This approach is particularly suitable for localization systems that rely on signal strength data, such as RSSI, as it effectively reduces the impact of distant reference points in environments with high measurement errors. By applying weighted processing, the influence of nearby reference points on the localization result is enhanced, ultimately improving the system’s accuracy and robustness.

### 2.3. Maximum Likelihood Estimation (MLE) for Indoor Localization

**Figure 4:**
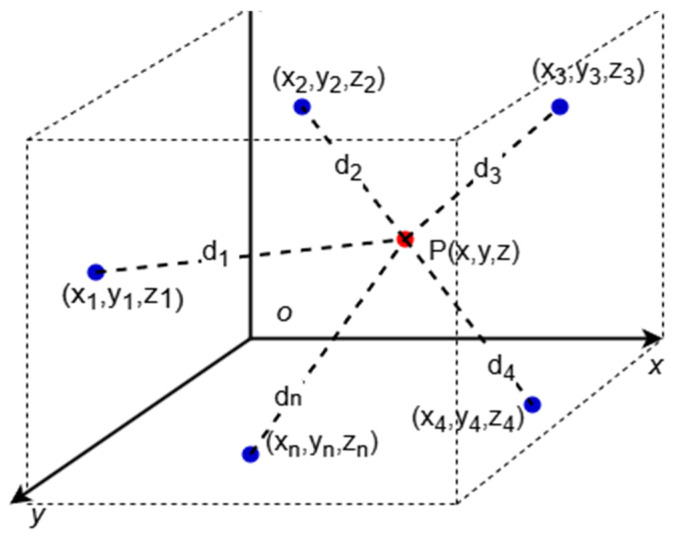
MLE schematic diagram.

The maximum likelihood estimation (MLE) localization model improves positioning accuracy by introducing a probabilistic model, especially when using RSSI as the ranging method. In this approach, assume that there are n anchor nodes used for localization, with coordinates $\left\{(x_{i},y_{i},z_{i})\}(i=1,2,\ldots ,n\right)$, and the corresponding distance measurement results are ${\hat{D}}_{i}(i=1,2,\ldots ,n)$. Figure 4 illustrates the schematic diagram of this localization model. Let the coordinate estimate of the blind node be Θ(x,y,z), then the conditional probability $P(\hat{D}|\Theta )$ represents the probability of observing the distance measurement vector $\hat{D}$ when the blind node’s coordinates are Θ. To obtain the maximum likelihood estimate of the blind node’s coordinates, we can calculate by maximizing the conditional probability, i.e.,:

(6) $$\hat{\Theta }=\arg\underset{\Theta }{\max\;}P\left(\hat{D}|\Theta \right),$$

Assume that the measurement errors follow a zero-mean Gaussian distribution, the likelihood function is:

(7) $$P\left(\hat{D}|\Theta \right)=\prod_{i=1}^{n}\;\frac{1}{\sqrt{2\pi \sigma_{i}^{2}}}\exp\left(-\frac{(\hat{d_{i}}-d_{i}{)}^{2}}{2\sigma_{i}^{2}}\right),$$

Here, ${\hat{d}}_{i}$ is the distance estimate obtained through RSSI, $d_{i}$ is the actual distance between the anchor node and the blind node, and $\sigma_{i}^{2}$ is the variance of the ith measurement error. Taking the logarithm of the likelihood function gives the log-likelihood function:

(8) $$\ln P\left(\hat{D}|\Theta \right)=-\frac{1}{2}\sum_{i=1}^{n}\left[\ln\left(2\pi \sigma_{i}^{2}\right)+\frac{(\hat{d_{i}}-d_{i}{)}^{2}}{\sigma_{i}^{2}}\right],$$

To obtain the optimal blind node position Θ(x,y,z), the error term $\sum_{i=1}^{n}\;\frac{({\hat{d}}_{i}-d_{i}{)}^{2}}{\sigma_{i}^{2}}$ needs to be minimized. Ultimately, the optimization problem is transformed into a least squares problem with the objective function:

(9) $$f\left(x,y,z\right)=\sum_{i=1}^{n}\;\beta_{i}{\left({\hat{d}}_{i}-\sqrt{(x-x_{i}{)}^{2}+(y-y_{i}{)}^{2}+(z-z_{i}{)}^{2}}\right)}^{2},$$

In the absence of weighting factors, the model degenerates into the least squares form:

(10) $$\sum_{i=1}^{n}\;{\left(\hat{d_{i}}-\sqrt{(x-x_{i}{)}^{2}+(y-y_{i}{)}^{2}+(z-z_{i}{)}^{2}}\right)}^{2},$$

By solving this least squares problem, the maximum likelihood estimate of the blind node’s position can be obtained. The above equation can be transformed into a system of linear equations:

(11) AX=B,

where *A* is the coefficient matrix of the linear equation system, and *B* is the constant term. The final solution is:

(12) $$X=(A^{T}A{)}^{-1}A^{T}B.$$

### 2.4. Theoretical Foundations and Relevance of Baseline Methods

The previous subsections have introduced three foundational localization algorithms, namely, 3D triangular centroid localization, 3D weighted centroid localization, and maximum likelihood estimation (MLE), which are widely used in wireless sensor network positioning. While these methods are not proposed as direct solutions to the specific challenges addressed in this work, they serve as theoretical baselines and conceptual references for the development and evaluation of the proposed NLW-MLE algorithm. To enhance clarity, this subsection outlines their methodological relevance and how they contribute to the structure and logic of the present study.

Specifically, the triangular centroid localization method provides a geometric benchmark for inferring target positions in 3D space under uniform signal strength assumptions. Its weighted extension incorporates static signal-based weighting, which conceptually relates to our use of RSSI and LQI metrics for spatial inference. Together, these two centroid-based approaches serve as comparative baselines to evaluate the added value of dynamic weighting and signal preprocessing strategies adopted in our method.

MLE, as discussed in Section 2.3, constitutes the statistical foundation of our approach. The proposed NLW-MLE algorithm can be regarded as an enhanced extension of classical MLE, incorporating multi-stage RSSI filtering and dynamic weight adjustments informed by link quality indicators. This progression represents a methodological evolution from static signal modeling to adaptive probabilistic estimation, aimed at improving robustness and accuracy in complex indoor environments.

These classical methods are further employed in the experimental section as benchmarks under identical test conditions. Their inclusion ensures consistency between theoretical grounding and empirical evaluation, and supports fair, interpretable performance comparisons. Overall, the discussion in Section 2 establishes the necessary algorithmic context for NLW-MLE, while also contributing to the methodological transparency and rigor of the study.

## 3. RSSI Preprocessing via Using Multi-Stage Filtering Techniques

In ZigBee-based positioning systems, RSSI measurements between nodes are frequently influenced by various environmental factors such as temperature, humidity, shadow fading, and multipath propagation. These effects result in RSSI signals that exhibit considerable fluctuations and noise. Since positioning calculations often rely directly on these unstable measurements, the resulting location estimates can be significantly affected by signal variability.

Moreover, RSSI values received by the nodes can vary substantially over time, and the presence of outliers due to random environmental interference is common. Such abnormalities not only degrade the reliability of wireless sensor network platforms but also lead to increased errors in distance estimation and localization.

To address these challenges, it is necessary to apply signal preprocessing techniques that can effectively enhance data quality before localization is performed. Filtering methods play a critical role in suppressing noise, identifying abnormal measurements, and stabilizing signal trends. In this section, we introduce a multi-stage RSSI filtering framework composed of several complementary techniques tailored to the characteristics of ZigBee signal environments.

Mean filtering [20] is widely adopted for its simplicity and computational efficiency, providing a basic level of noise suppression. Dixon’s Q test is useful for identifying and eliminating statistical outliers, thereby improving the accuracy of RSSI-based measurements. Gaussian filtering contributes to further signal smoothing by applying a weighted averaging scheme, while Kalman filtering enables dynamic state estimation by modeling system uncertainty. Together, these techniques form a multi-stage preprocessing framework that significantly enhances the robustness of localization systems under real-world signal conditions. Prior research confirms that combining multiple filtering strategies is crucial to improving positioning accuracy in RSSI-based indoor localization scenarios.

### 3.1. Mean Filtering

The mean filtering method processes the set of RSSI data received by the node through statistical operations to generate a new sequence of data points. Typically, the length of the data set is fixed, and when a new data point is added, the oldest data point is removed, dynamically updating the data set.

During this process, all data points are considered equally weighted, with no prioritization or differentiation in importance. Therefore, a moving average filtering method can be directly applied. Its mathematical expression is given by:

(13) $$RSSI=\;\frac{1}{n}\sum_{i=1}^{n}\;RSSI_{i},$$

where RSSI represents the received signal strength, *n* is the number of signal strength samples, and $RSSI_{i}$ denotes the signal strength value of the ith sample.

Although Equation (13) defines RSSI as the average of *n* received signal samples, the value of *n* in our implementation is fixed and kept relatively small (typically 10–20 samples). These samples are collected at the ZigBee module’s native transmission rate (10–50 Hz), resulting in a total acquisition time of only a few hundred milliseconds. This level of delay is negligible in the context of most static or semi-dynamic indoor localization tasks.

Moreover, signal preprocessing including the mean filtering step is executed on a host computer or control terminal after data collection, rather than on embedded sensor nodes. This architectural design offloads computational complexity from resource-constrained ZigBee devices, ensuring that processing latency does not interfere with real-time communication or data acquisition.

We acknowledge that in latency-critical applications such as human–robot interaction or real-time navigation–response delay may be a more prominent concern. In such cases, adaptive filtering schemes with tunable sample windows or predictive estimation strategies may be explored in future work to further minimize response time without compromising stability.

### 3.2. Dixon’s Q Test for Outlier Detection

Dixon’s Q test is a statistical method used to identify and eliminate outliers in a dataset, playing a crucial role in improving the accuracy of distance measurements. This is particularly important when using RSSI data for distance estimation, as the presence of outliers can significantly affect the accuracy of the results.

The steps for applying Dixon’s Q test to filter RSSI values are as follows:

Step 1: Collect n RSSI data points and arrange them in ascending order as $RSSI_{1}\leq RSSI_{2}\leq RSSI_{3}\leq \cdots \leq RSSI_{n}$, then determine the significance level α=0.05.

Step 2: Based on the sample size (30 in this case), use Dixon’s Q test formula to check for outliers at both ends of the dataset. For sample sizes between 14 and 30, the formula is:

(14) $$D_{n}\;=\;\frac{x_{\left(n\right)}-x_{\left(n-1\right)}}{x_{\left(n\right)}-x_{\left(3\right)}}\;{D_{n}}^{\prime }\;=\;\frac{x_{\left(3\right)}-x_{\left(1\right)}}{x_{\left(n-2\right)}-x_{\left(1\right)}},$$

where $D_{n}$ is the test statistic for detecting high-end outliers, and ${D_{n}}^{\prime }$ is the test statistic for detecting low-end outliers.

Step 3: In this study, a significance level of α=0.05 is chosen, and the corresponding critical value $D\left(\alpha ,n\right)$ is obtained from Dixon’s Q test critical value table.

Step 4: Compare the calculated Dixon’s Q test statistic with the critical value. If the Dixon’s Q test statistic exceeds the critical value, the dataset is determined to contain outliers.

Step 5: Remove the detected outliers and repeat the above steps using the remaining RSSI values until no more outliers exist in the dataset.

### 3.3. Filtering for RSSI Smoothing

By constructing a Gaussian distribution model based on multiple RSSI values received by the same node, this method filters RSSI values by selecting those with high probability as valid data according to the characteristics of the Gaussian distribution.

The measurement of signal amplitude follows the probability density function of a Gaussian distribution, which is given by:

(15) $$\phi \left(x\right)\;=\;\frac{1}{\sqrt{2\pi }\sigma }e^{-\frac{(x-\mu {)}^{2}}{2\sigma^{2}}},$$

where:

(16) $$\mu =\;\frac{1}{n}\sum_{i=1}^{n}\;x_{i},$$

(17) $$\begin{array}{c} \sigma =\sqrt{\frac{1}{n-1}\sum_{i=1}^{n}\;{\left(x_{i}-\mu \right)}^{2}} \end{array}.$$

Gaussian filtering is a widely used signal processing technique, particularly suitable for handling noisy and highly fluctuating signals. By applying a weighted average calculation to the signal, this method effectively reduces noise interference while enhancing signal accuracy and stability.

However, Gaussian filtering has certain limitations. Its performance depends on the number of sample data points, and when the sample size is insufficient, it may not significantly reduce noise effects. Additionally, it has limited effectiveness against persistent interference, such as energy reflections or shadowing effects. Furthermore, if signal processing takes too long, it may negatively impact the real-time performance of the system. Therefore, in practical applications, parameters should be adjusted based on specific scenarios to achieve a proper balance between noise reduction and system response speed.

In this study, a probability range of 0.68 ≤ *ϕ*(*x*) ≤ 1 was selected for analyzing RSSI signal strength values. Choosing this range means focusing on RSSI values within ±1σ of the mean, which includes the most representative data points, as they are tightly distributed around the overall average. Gaussian filtering effectively captures consistent and dominant signal strength characteristics in the environment while ignoring outliers that may result from sudden environmental fluctuations, device errors, or other random anomalies.

### 3.4. Kalman Filtering for Dynamic Signal Estimation

Kalman filtering is a recursive method used to estimate the state of a dynamic system. By combining the system model and observational data, Kalman filtering continuously updates the state estimate and state covariance matrix, achieving optimal estimation. This method performs exceptionally well in dynamic and frequently changing environments for signal processing. By simplifying and optimizing the algorithm, such as using efficient data structures and workflows, the filter can operate efficiently and stably.

Kalman filtering consists of two main stages: the prediction stage and the update stage.

In the prediction stage, the system state is estimated using the dynamic model, with the following equations:

1. State Prediction:

(18) $$x_{k}=A_{k}\cdot x_{k-1}+B_{k}\cdot u_{k},$$

where $x_{k}$ represents the estimated state at time step k, $A_{k}$ is the state transition matrix, $B_{k}$ is the control input matrix, and $u_{k}$ is the control input.

2. Error Covariance Prediction

This equation predicts the error covariance, where $P_{k}$ is the current error covariance matrix, and $Q_{k}$ represents the process noise covariance matrix.

In the update stage, the prediction is combined with the observed data by computing the Kalman gain, which is then used to correct the state and error covariance using the following equations.

3. Computation of the Kalman Gain

(19) $$K_{k}\;=\;P_{k}\cdot H_{k}^{T}\cdot (H_{k}\cdot P_{k}\cdot H_{k}^{T}+R_{k}{)}^{-1},$$

where $H_{k}$ is the observation matrix, and $R_{k}$ is the observation noise covariance matrix.

4. State Correction

(20) $$x_{k}=x_{k}+K_{k}\cdot \left(z_{k}-H_{k}\cdot x_{k}\right),$$

where $z_{k}$ represents the observed measurement.

5. Error Covariance Correction

(21) $$P_{k}\;=\;\left(I-K_{k}\cdot H_{k}\right)\cdot P_{k},$$

where I is the identity matrix. Equations (18)–(21) form the core of the Kalman filter, which recursively estimates the state by combining predictions with observed data, thereby improving the accuracy of state estimation and error correction.

### 3.5. KDGM: A Combined Filtering Framework

First, the Kalman filtering algorithm is applied to the initial RSSI values. Kalman filtering is a recursive filtering technique that reduces noise and uncertainty by continuously estimating and correcting the system state, thereby improving the accuracy and stability of the data.

Next, the Dixon filtering method is applied to the Kalman-filtered RSSI values. At a significance level of α=0.05, the computed Dixon statistic is compared with the critical value until no more outliers exist in the dataset. Dixon filtering is a statistical method that identifies and removes outliers by calculating the deviation of sample data from the median and comparing it with other samples, significantly reducing data errors and biases.

Then, the Gaussian filtering algorithm is used to smooth the Dixon-filtered RSSI values and select values within the probability range of $0.68\leq F_{RSSI}\leq 1$. Gaussian filtering, based on Gaussian distribution, smooths data through weighted averaging or convolution operations, effectively eliminating noise and sudden interference while improving data stability and reliability.

Finally, the Gaussian-filtered RSSI values undergo accumulation and averaging operations to obtain the final RSSI value. This accumulation and averaging process further enhances the accuracy and stability of the data, reducing measurement errors and providing a more precise reflection of wireless signal strength and propagation distance.

## 4. Proposed Localization Method: NLW-MLE

The previous sections reviewed several representative localization techniques, including centroid-based algorithms and standard likelihood estimation approaches. While these methods have demonstrated effectiveness in ideal or low-noise environments, their performance may degrade significantly in the presence of unstable wireless signals and irregular multipath effects. Based on these limitations, this section presents an extension of the classical maximum likelihood estimation (MLE) model, incorporating dynamic signal quality awareness and multi-source error mitigation into the estimation process.

MLE is a robust probabilistic localization technique widely adopted in wireless sensor networks. Combining RSSI with LQI measurements enhances the MLE approach, providing superior accuracy and robustness under diverse environmental conditions [21]. Moreover, recent studies have demonstrated that applying adaptive weight optimization strategies to the likelihood estimation process can further enhance localization performance by accounting for measurement uncertainty and varying signal quality.

In practical applications, ranging errors are influenced not only by noise but also by link quality. To improve positioning accuracy, this method introduces a noise and LQI weighting mechanism into the traditional MLE positioning method to enhance location estimation. Specifically, the traditional MLE method estimates the target position by minimizing the sum of squared ranging errors. However, in the presence of high noise levels and unstable signal quality, this approach can be significantly affected by ranging data with large errors. To address this issue, we dynamically adjust the weight of each ranging measurement based on its noise level and LQI. In this method, ranging values with lower noise levels and higher LQI are considered more reliable and are assigned higher weights. Conversely, ranging values with higher noise levels or lower LQI contribute less to location estimation and are assigned lower weights. This weighting mechanism effectively reduces the impact of noisy or low-quality ranging data on the final position estimate, thereby improving positioning accuracy.

To implement this weighting mechanism, we first compute the weight for each ranging measurement. The weight calculation process integrates two key factors: ranging noise level and LQI value. Ranging values with low noise and high LQI receive higher weights, while those with high noise and low LQI are assigned lower weights. Finally, the weight of each ranging measurement is dynamically adjusted based on the combined influence of noise and LQI, leading to a weighted final position estimate.

### 4.1. Dynamic Weight Calculation Based on Noise and LQI

To dynamically adjust the weights based on ranging noise and LQI, the noise weight and LQI weight are first computed separately and then combined to form the final weighted value.

Assuming there are *N* anchor nodes, the ranging distance between the blind node and the ith anchor node is denoted as $d_{i}$, the noise level as $\sigma_{i}$, and LQI as $L_{i}$. The weight calculation process is as follows:

1. Noise Weight

The lower the noise level, the more reliable the ranging result. Therefore, the noise weight can be expressed as the inverse square of the ranging noise. The specific formula is:

(22) $$w_{noise,i}=\frac{1}{\left(\sigma_{i}^{2}+\epsilon )\cdot (d_{i}^{2}+\epsilon \right)},$$

where $\sigma_{i}$ is the noise level of the iith anchor node, $d_{i}$ is the ranging distance between the blind node and the ith anchor node, and ϵ is a small constant used to avoid division by zero errors.

2. LQI Weight

LQI reflects the quality of the signal, namely, the higher the LQI, the better the signal quality. To adjust the weight based on link quality, the LQI values of all anchor nodes are first normalized to ensure they fall within the range [0,1]. The LQI weight is then calculated using the following formula:

(23) $$w_{i}=\frac{1}{\left(\sigma_{i}^{2}+\epsilon )\cdot (d_{i}^{2}+\epsilon \right)}\cdot \frac{L_{i}}{\max\left(L\right)},$$

3. Comprehensive Weight

By combining the noise weight and the LQI weight, the final weight for each anchor node is obtained. The comprehensive weight is calculated as the product of the noise weight and the LQI weight, given by the following formula:

(24) $$w_{i}=\frac{1}{\sigma_{i}^{2}+\epsilon }\cdot \frac{L_{i}}{\max\left(L\right)},$$

4. Weighted Sum and Normalization

To ensure that the sum of the weights equals 1 and to prevent extreme weights from excessively influencing the result, the final comprehensive weighted value is calculated through weighting followed by normalization, given by the following formula:

(25) $$w_{i}^{\prime }=\frac{w_{i}\cdot w_{LQI,i}}{\sum_{i=1}^{N}\;w_{i}\cdot w_{LQI,i}},$$

This step ensures that the sum of all weights equals 1, preventing any single weight from having an excessive or insufficient impact on the position estimation. This improves the stability and accuracy of the calculation.

By applying the above formulas, all weights satisfy the normalization requirement, ensuring that they do not introduce any unreasonable influence on the final positioning result.

### 4.2. Formulation of the Weighted Error Function

The weighted error function is obtained by summing the weighted ranging error terms. The specific formula is given by:

(26) $$f\left(x,y,z\right)=\sum_{i=1}^{n}\;w_{i}{\left[(x_{i}-x{)}^{2}+(y_{i}-y{)}^{2}+(z_{i}-z{)}^{2}-d_{i}^{2}\right]}^{2},$$

where $w_{i}$ is the comprehensive weight of the ith reference point, representing its contribution to the position estimation.

By minimizing the weighted error function f(x,y,z), the estimated coordinates of the blind node are obtained. The specific linear equation system follows a structure similar to the standard MLE method, with the difference that the error terms in the equations are adjusted according to the assigned weights.

Finally, the estimated coordinates of the blind node $\hat{X}$ are determined using the following formula:

(27) $$\hat{X}=(A^{T}A{)}^{-1}A^{T}b\;$$

By using the NLW-MLE method, we can achieve more accurate positioning results while accounting for the effects of noise and link quality.

## 5. Experimental Evaluation and Results

### 5.1. Simulation Setup

This study uses MATLAB (R2020b) as the simulation tool to conduct a simulation for the three-dimensional positioning problem. During the simulation, 50 blind nodes are randomly generated and simulated within the three-dimensional space of a room. The simulation parameter settings are as follows: the anchor node positions include six anchors located at (0, 0, 0), (5, 0, 0), (0, 5, 0), (0, 0, 5), (2.5, 2.5, 5), and (5, 5, 5). Each unknown node performs ranging measurements with each anchor node, with each measurement repeated 80 times to account for measurement variations.

In the simulation, the parameters of the path loss model are set as follows: $P_{0}=-40.41,n=2.05,X_{\sigma }=3.9$. Additionally, the simulation considers the effects of Rician fading, where the K-factor is set to 1.79, and dynamic obstacles, with a fading coefficient of σ=0.9. These parameters, combined with the path loss model, simulate the positioning accuracy under different channel conditions, adjusting the ranging error through different RSSI values and noise levels.

Additionally, four positioning methods are compared, namely the triangular centroid method, the triangular weighted centroid method, MLE, and NLW-MLE. Each method calculates the positioning error separately and performs statistical analysis. The errors record the positioning accuracy of each method, ultimately outputting the mean error and standard deviation of different positioning methods.

### 5.2. Simulation Results and Discussion

To verify the accuracy and robustness of the NLW-MLE positioning algorithm, the triangular centroid method (TCM), triangular weighted centroid method (TWCM), and MLE were compared with the proposed NLW-MLE algorithm. The simulation experiments were conducted using the parameter settings described in the Section 5.1, yielding positioning results for 50 blind nodes. Some of the experimental results are presented in Table 1 and Table 2.

From Table 1 and Table 2, it can be observed that the triangular centroid method (TCM) and triangular weighted centroid method (TWCM) exhibit relatively large positioning errors. Comparing MLE with the proposed NLW-MLE algorithm, the positioning error of NLW-MLE is much lower than that of MLE at certain data points, providing more accurate results. The positioning accuracy of NLW-MLE is improved by 11.7% compared to traditional MLE.

To further highlight the superiority of the NLW-MLE positioning algorithm, its performance across different data points is analyzed using a heatmap, line graph, and 3D positioning result visualization.

**Figure 5:**
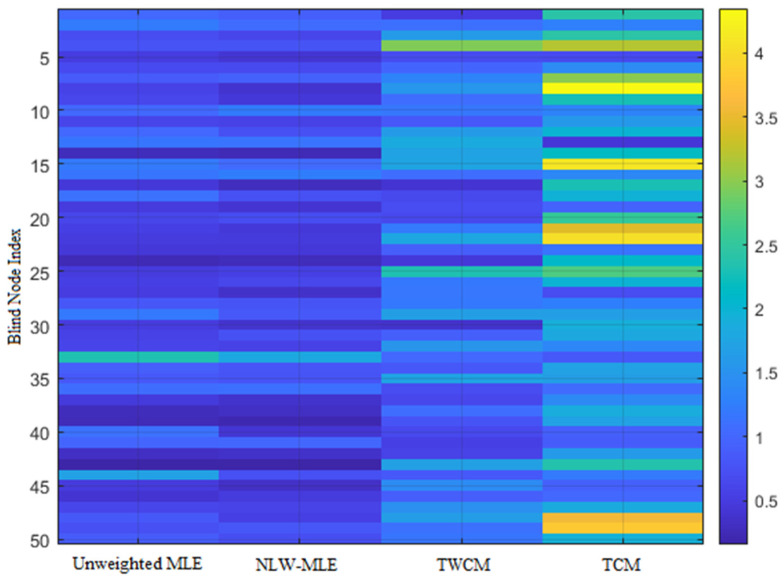
Heatmap of positioning errors for different methods.

As shown in Figure 5, the heatmap clearly illustrates that NLW-MLE achieves positioning errors below 2 m for most nodes, with a uniform error distribution, demonstrating its advantage in positioning accuracy. In contrast, TCM and TWCM show generally higher errors, with significant fluctuations at certain nodes, indicating their limitations in terms of accuracy and stability.

**Figure 6:**
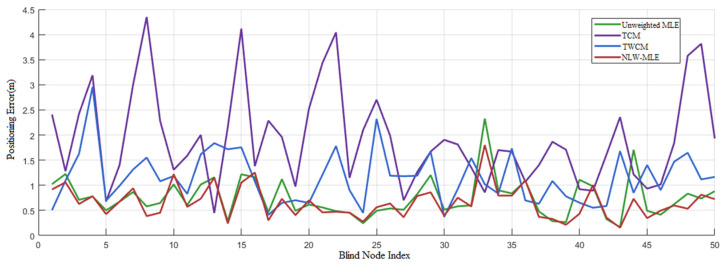
Comparison of positioning errors across different methods.

The line graph in Figure 6 compares the error variation trends among different positioning algorithms. It is evident that NLW-MLE has the lowest and most stable positioning error, maintaining a consistent error range between 0.5 and 1 m. In comparison, MLE exhibits significantly higher errors than NLW-MLE at blind node indices 1, 8, 17, 33, and 44.

**Figure 7:**
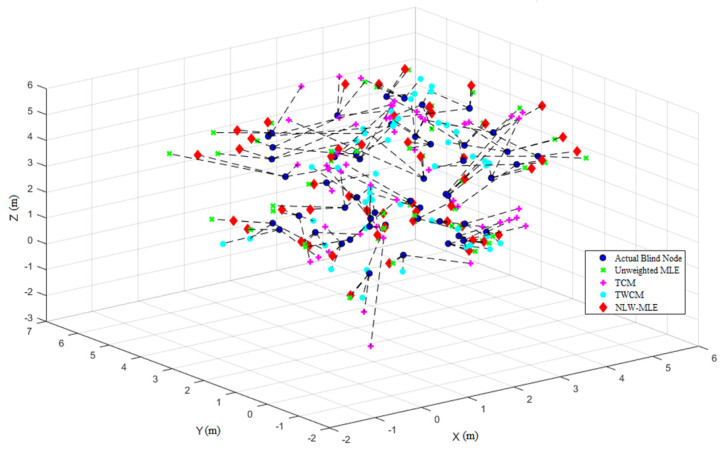
3D positioning result visualization for different methods.

Finally, the 3D positioning result visualization in Figure 7 provides a comparative analysis of the positioning accuracy and distribution across different methods, offering a clearer understanding of the precision and stability of the NLW-MLE algorithm.

### 5.3. Experimental Validation in a Simulated Fuel Depot Environment

**Figure 8:**
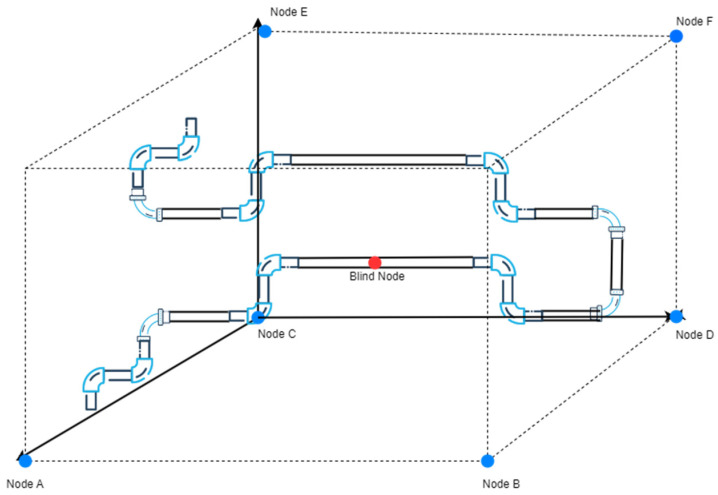
Schematic diagram of the simulated environment.

The software testing of the aviation fuel pipeline leak point localization system was conducted in Laboratory 1228 of the university. The reasons for choosing this laboratory as the test environment are as follows: the non-line-of-sight (NLOS) conditions within the laboratory can effectively simulate the actual situation of pipeline obstruction in fuel depots. In real fuel depots, due to the structure and layout of pipelines, obstructions may occur, which can affect wireless signal propagation and lead to changes in positioning accuracy. Therefore, the indoor NLOS environment provides a suitable testing scenario for the system, allowing for a realistic evaluation of its performance in actual fuel depot conditions. The coordinates of the anchor nodes used in this test are: (0, 0, 0), (1.8, 0, 0), (0, 3, 0), (1.8, 3, 1.5), (0, 3, 1.5), and (1.8, 3, 0). Since it was not possible to accurately capture the layout of each node in photographs, a 3D schematic diagram was drawn instead, in which all anchor nodes are marked with blue dots, as shown in Figure 8, which illustrates the simulated test environment.

In the experiment, several positioning points within the coverage area of the anchor nodes were first selected, and their actual positions were measured. Then, the blind node was placed at these positioning points sequentially, and the system’s measurement results were obtained from the upper computer interface. Next, the actual positions of the positioning points were compared with the system’s measured positions and recorded. The final experimental positioning results are shown in Table 3.

**Figure 9:**
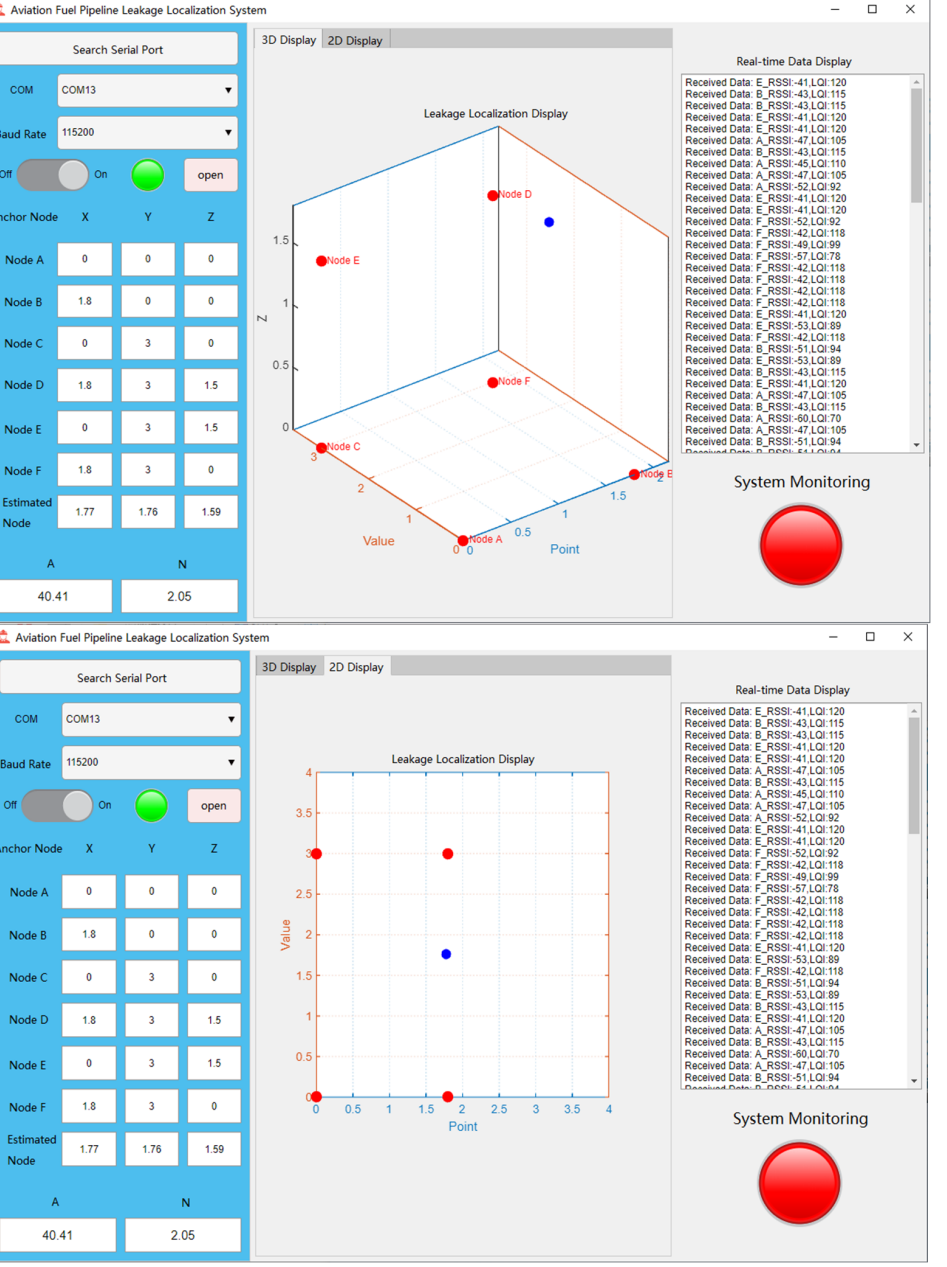
Alarm diagram on the upper computer interface.

Figure 9 illustrates the system’s response upon detecting volatile gases: the monitoring status indicator changes from green to red, signaling an alarm state. At the same time, a blue dot clearly appears on the coordinate axis of the upper computer interface, indicating the actual positioning coordinates to help users locate the leakage point. Additionally, the system supports displaying the positioning results in both three-dimensional space and two-dimensional plane, providing more intuitive information to assist users in accurately identifying the leakage location. From Table 3, it can be seen that the actual positioning error is significantly higher than the positioning error in the simulation. The real-world results indicate that the system successfully achieves 3D positioning of the blind node. However, the positioning error is larger compared to the simulation results. The main sources of this error include the following aspects:

(1) Antenna Error Impact

The installation position of each node’s antenna may cause variations in signal strength across different directions and areas. When the signal strength is uneven, the received signal strength at the receiver is affected, impacting the accuracy of the positioning algorithm. In real-world environments, due to the directional characteristics and propagation properties of the antenna, signals may experience attenuation or reflection, leading to positioning errors. Additionally, if the antenna’s directionality and radiation pattern are not optimal, misalignment or deviation may result in weak or distorted signals, causing inaccurate distance measurements. This error is particularly pronounced in complex environments with obstacles or walls, which can cause the positioning results to deviate from the actual position.

(2) Positioning Range and Node Deployment

In practical applications, the deployment of anchor nodes may not be as ideal as in simulations. In simulations, nodes are typically placed in optimal positions to ensure good coverage and high positioning accuracy. However, in real-world scenarios, suboptimal deployment or signal obstruction may lead to positioning errors.

### 5.4. Planned Field Deployment and Practical Constraints

To support real-world validation, this study originally planned to deploy the proposed NLW-MLE system in an operational aviation fuel depot at a civil aviation flight training airport. The target site provides a highly representative environment with dense metal pipelines, structural occlusions, and complex signal propagation conditions commonly encountered in practical applications.

However, due to safety regulations governing electronic equipment deployment in civil aviation fuel storage facilities, field testing requires formal approval and strict site clearance procedures. At the time of this study, the approval process was still ongoing. As a result, only preliminary site investigations and environmental documentation were completed.

**Figure 10:**
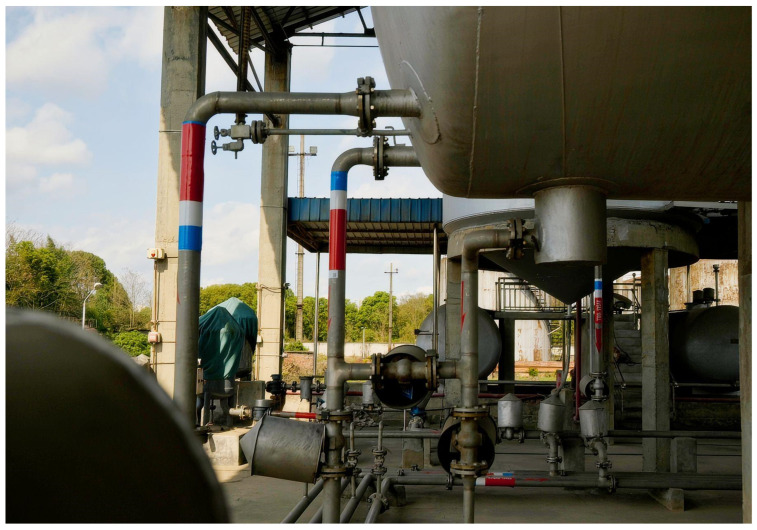
Planned deployment site inside an aviation fuel depot at a civil aviation flight training airport.

Figure 10 presents an interior view of the intended deployment site, highlighting the metal enclosures and compact pipeline layout that are expected to influence wireless signal behavior. Upon obtaining the necessary authorization, the system will be deployed and evaluated on-site as part of the next phase of this research project.

## 6. Discussion

Although several recent localization approaches have been cited in this study, including AOA, TOA, and TDOA based methods [3,4,5,6,7], direct experimental comparisons with these works were not performed due to fundamental differences in signal models, hardware assumptions, and implementation platforms. For example, the AOA based techniques in [3,4] require antenna arrays to estimate signal arrival angles, while the TOA and TDOA methods in [5,6,7] depend on precise time synchronization and timestamping modules. These hardware configurations are incompatible with the ZigBee based system used in our study, which relies on low-power RSSI and LQI measurements without external synchronization or directional antennas. This makes direct comparison with those methods technically infeasible within our hardware and software constraints.

Furthermore, the evaluation environments and performance metrics used in these studies differ substantially, with some focusing on RFID systems or ultra-wide band (UWB) based platforms, and others employing distinct signal processing pipelines or assumptions about network topology and device capabilities. Due to these discrepancies, directly replicating or benchmarking such methods within our experimental setup may not yield fair or interpretable comparisons.

Given these constraints, we focused our evaluation on classical localization baselines such as MLE and centroid based methods that share a similar signal acquisition and deployment context. This allows for a more controlled and meaningful assessment of the improvements introduced by the proposed multi-stage filtering and LQI-aware weighting strategy. Future work will explore integrating additional signal modalities or simulation-based comparisons to broaden the scope of performance benchmarking under diverse technical conditions.

In addition, the current evaluation is based on a limited set of representative test points in both the simulation and semi-physical environments. These points were selected to capture typical localization scenarios, including central, edge, and non-line-of-sight (NLOS) conditions. Although additional test positions were explored during the development phase, only a subset was presented in the manuscript for clarity, interpretability, and space considerations.

We acknowledge that a broader distribution of test points and more comprehensive statistical metrics such as cumulative distribution functions (CDFs) could further enhance the analysis. However, given the limited scale of the current deployment and the sparsity of available real-world positions, we chose to prioritize robust pointwise comparisons over statistical representations that may not be fully supported by the dataset. We plan to include expanded test coverage and CDF based evaluation in future work when larger-scale field deployments become feasible. Such extensions will also enable more robust statistical modeling and comprehensive algorithmic benchmarking.

## 7. Conclusions

This study presents a refined three-dimensional indoor localization method, NLW-MLE, which extends the conventional maximum likelihood estimation framework by integrating multi-stage RSSI signal preprocessing and a dynamic weighting mechanism based on noise level and link quality indicator (LQI). The proposed approach aims to improve positioning robustness under practical constraints such as multipath propagation, signal fluctuations, and anchor placement irregularities.

Through simulation and semi-physical experiments, the NLW-MLE method demonstrated improved localization accuracy and stability compared to classical MLE and centroid based algorithms. The integration of filtering techniques, namely, Kalman, Dixon’s Q, and Gaussian smoothing helped reduce the impact of outliers and environmental noise, contributing to more reliable ranging inputs. Evaluation results also showed that the algorithm maintained relatively consistent performance across different spatial conditions.

While the current evaluation is based on a limited set of representative test points, the observed improvements suggest that the proposed method may be applicable in resource-constrained wireless sensor network scenarios such as indoor monitoring and infrastructure sensing. Future work will focus on expanding the evaluation scope, exploring support for dynamic target tracking, and adapting the approach to other wireless platforms, including UWB and Bluetooth-based systems.

## Figures and Tables

**Table 1:** Comparison of triangular centroid and weighted centroid methods.

Actual Coordinates	Triangular Centroid Method		Triangular Weighted Centroid Method	
	Positioning Results	Positioning Error (m)	Positioning Results	Positioning Error (m)
(0.2, 0.57, 2.23)	(0.36, −0.69, 4.28)	2.41	(0.44, 0.44, 2.66)	0.5
(2.74, 4.7, 4.14)	(3.13, 5.22, 5.24)	1.28	(3.33, 3.85, 4.48)	1.09
(2.05, 2.98, 3.51)	(2.44, 2.44, 5.84)	2.42	(2.48, 2.48, 5)	1.63
(4.41, 4.4, 1.18)	(3.02, 6.65, 2.96)	3.19	(2.97, 4.21, 3.76)	2.96
(1.36, 2.08, 4.41)	(1.37, 2.69, 4.13)	0.68	(1.95, 1.95, 4.75)	0.69
(2.84, 1.89, 4.28)	(2.13, 2.13, 5.47)	1.4	(2.43, 2.43, 5)	0.99
(1.45, 4.65, 0.26)	(0.41, 1.9, 0.86)	3	(0.14, 4.53, 0.29)	1.31
(3.79, 2.72, 1.54)	(3.94, 0.69, 5.39)	4.35	(4.14, 2.02, 2.88)	1.55
(1.48, 0.22, 3.12)	(0.73, 2.1, 4.18)	2.28	(0.76, 0.76, 3.73)	1.08
(4.16, 1.38, 3.7)	(3.6, 1.91, 4.75)	1.31	(3.56, 2.4, 3.83)	1.18
(0.34, 0.15, 2.89)	(0.38, 0.18, 4.47)	1.59	(0.44, 0.44, 3.66)	0.83
(0.9, 4.08, 3.46)	(2.06, 3.13, 4.78)	2	(2.41, 3.6, 3.8)	1.62
(2.93, 2.42, 1.12)	(2.65, 0.89, 2.62)	2.16	(2.67, 0.78, 1.56)	1.72
(3.91, 2.97, 1.43)	(4.14, 1.22, 5.15)	4.12	(4.21, 2.18, 2.96)	1.75
(4.6, 2.47, 3.77)	(3.92, 3.66, 3.98)	1.38	(3.78, 3, 4.22)	1.08

**Table 2:** Comparison of positioning results between MLE and NLW-MLE algorithms.

Actual Coordinates	MLE		NLW-MLE	
	Positioning Results	Positioning Error (m)	Positioning Results	Positioning Error (m)
(0.2, 0.57, 2.23)	(−0.81, 0.5, 2.39)	1.02	(−0.71, 0.55, 2.34)	0.92
(2.74, 4.7, 4.14)	(3.6, 5.09, 4.92)	1.22	(3.11, 4.99, 5.09)	1.06
(2.05, 2.98, 3.51)	(1.52, 3.12, 3.96)	0.71	(1.68, 3.11, 4)	0.63
(4.41, 4.4, 1.18)	(4.5, 4.57, 1.93)	0.78	(4.46, 4.57, 1.94)	0.78
(1.36, 2.08, 4.41)	(1.14, 1.75, 4.72)	0.5	(1.38, 1.95, 4.82)	0.43
(2.84, 1.89, 4.28)	(2.23, 1.67, 4.46)	0.67	(2.27, 1.78, 4.63)	0.68
(1.45, 4.65, 0.26)	(1.37, 5.45, −0.07)	0.87	(1.35, 5.52, -0.09)	0.94
(3.79, 2.72, 1.54)	(3.93, 2.72, 0.98)	0.58	(3.83, 2.68, 1.16)	0.39
(1.48, 0.22, 3.12)	(1.03, −0.24, 3.21)	0.65	(1.17, −0.1, 3.22)	0.45
(4.16, 1.38, 3.7)	(4.75, 0.74, 4.22)	1.02	(4.82, 0.6, 4.36)	1.22
(0.34, 0.15, 2.89)	(0.26, −0.41, 3.09)	0.61	(0.3, −0.38, 3.09)	0.57
(0.9, 4.08, 3.46)	(0.08, 4.6, 3.76)	1.01	(0.41, 4.39, 3.91)	0.73
(2.93, 2.42, 1.12)	(2.98, 2.6, 0.9)	0.29	(2.94, 2.59, 0.96)	0.24
(3.91, 2.97, 1.43)	(4.8, 3.77, 1.2)	1.22	(4.75, 3.61, 1.39)	1.05
(4.6, 2.47, 3.77)	(5.56, 3.03, 4.09)	1.38	(5.7, 2.54, 4.35)	1.25

**Table 3:** Experimental positioning results.

Actual Coordinates	Positioning Results	Positioning Error (m)
(0, 2, 1)	(−0.56, 0.80, 1.28)	1.35
(1, 2, 0)	(1.21, 1.83, 0.98)	1.01
(1, 0, 1)	(1.87, −0.47, 0.18)	1.28
(1, 1, 0)	(0.19, 0.84, 0.66)	1.05
(1, 1, 1)	(1.98, 0.17, 1.62)	1.42
(1, 3, 1)	(1.61, 1.79, 2.03)	1.7
(0, 2, 1)	(1.01, 2.73, 1.37)	1.3
(1, 3, 0)	(1.55, 2.34, 0.82)	1.18

## Data Availability

The data used to support the findings of this study are available from the corresponding author upon request.
